# Roles of cytokines in modulating *Trypanosoma brucei rhodesiense* infection outcomes in vervet monkeys

**DOI:** 10.3389/fpara.2025.1725651

**Published:** 2026-01-12

**Authors:** Clarah Jebet, John Kibuthu Thuita, Daniel Masiga, Benedict Owino Orindi, John Oidho, Mark C. Field, Enock Matovu, Vincent Owino Adung’a

**Affiliations:** 1 Department of Biochemistry and Molecular Biology, Egerton University, Nakuru, Kenya; 2 Biotechnology Research Institute - Kenya Agricultural and Livestock Research Organisation, Chemotherapy Division, Primate Section, Kikuyu, Kenya; 3 Department of Animal Sciences, School of Agriculture and Food Sciences, Meru University of Science and Technology, Meru, Kenya; 4 Department of Molecular Biology, International Centre of Insect Physiology and Ecology, Nairobi, Kenya; 5 Department of Public Health and Primary Care, Leuven Biostatistics and Statistical Bioinformatics Centre, Leuven, Belgium; 6 School of Life Sciences, University of Dundee, Dundee, United Kingdom; 7 Institute of Parasitology, Czech Academy of Sciences, České Budějovice, Czechia; 8 Department of Biomedical Laboratory Technology and Molecular Biology, College of Veterinary Medicine, Animal Resources and Biosecurity (COVAB), Makerere University, Kampala, Uganda

**Keywords:** African trypanosomiasis, cytokines, immune response, Immunomodulation, Infection, *Trypanosoma brucei* rhodesiense

## Abstract

**Introduction:**

Human African trypanosomiasis (HAT), caused by *Trypanosoma brucei rhodesiense*, is categorized as acute due to rapid disease progression but presents varying clinical outcomes. Although the mechanisms underpinning differential clinical progression are poorly understood, both host and parasite factors are implicated. Therefore, we sought to elucidate roles of primate host factors in mediating varying *T. b. rhodesiense* infection outcomes.

**Methods:**

Here, we assessed the roles of selected host cytokines in disease progression using a tsetse-mediated infection in a non-human primate (NHP) vervet monkey model that closely mimics HAT and natural infection. We quantified eight cytokines, including TNF-α, IFN-γ, IL-10, IL-6, IL-12, and IL-1β, as well as the brain injury biomarker S100b and clinical data, and compared acute and chronic infections. In addition,

**Results:**

Monkeys infected with KETRI 3801 and KETRI 3928 had mean survival times of 28 and 95 days, respectively. In both infected groups, cytokine levels were significantly higher than those in uninfected controls (p < 0.05). IL-12, IL-6, and IL-1β cytokines were significantly elevated (p < 0.05) from early-stage disease to the onset of late-stage disease. IL-1β, IL-6, IL-12, and IL-10 are implicated in pro- and counter inflammatory responses. In addition, cerebrospinal fluid parasite and white blood cell levels were higher in KETRI 3801 infections compared with KETRI 3928 infections.

**Discussion:**

We conclude that cytokines play roles in modulating disease progression and severity in an NHP model of HAT, which is important for understanding varying infection outcomes.

## 1 Introduction

Human African trypanosomiasis (HAT), or colloquially sleeping sickness, is a neglected tropical disease (NTD). HAT is caused by two subspecies of the protozoan parasite *Trypanosoma brucei*: *T. b. gambiense*, endemic in central and west Africa, and *T. b. rhodesiense*, endemic in east and southern Africa. Both are transmitted by blood-sucking tsetse flies (genus *Glossina*). HAT is a focal disease endemic in 36 countries of sub-Saharan Africa and affects some of the world’s poorest populations, with an estimated 55 million people at risk of infection and three million living in moderate- to high-risk areas (Simarro et al., 2010; Franco et al., 2022; WHO, 2023). However, the number of HAT cases reported in 2019 and 2020 was 992 and 663, respectively, indicating that human disease has come under control (Franco et al., 2022). During 2017–2018, reported Rhodesian HAT represented 2% of total HAT cases, while in 2018–2020, Rhodesian HAT represented 13% of the total number (Franco et al., 2020, 2022). Therefore, despite highly successful campaigns to reduce case numbers, HAT remains a public health threat in sub-Saharan Africa.

Rhodesian HAT is a zoonotic disease with livestock and wildlife as reservoirs. The disease has been reported in non-endemic countries, imported through travelers visiting areas of high transmission; among the latest was a case from India (Shah et al., 2022). The number of cases from non-endemic regions during the 2011–2020 period was 16, with 71% of the cases caused by *T. b. rhodesiense* and 29% by *T. b. gambiense (*Franco et al., 2022). Rhodesian HAT is categorized as acute, with fast progression to late stage and ultimate death if not detected and treated early.

Previous pathogenicity studies of *T. b. rhodesiense* isolates have indicated variation in disease progression and virulence. Recently, differences in survival times with various categories of pathogenicity have been described from small rodent model studies (Ndung’u et al., 2020; Limo et al., 2021), consistent with findings that clinical Rhodesian HAT virulence increases northwards within the eastern and southern African endemic region, even though the rodent infection model is far removed from human infections. Significantly, patients manifest acute and chronic infection, despite all being infected with strains of *T. b. rhodesiense* confirmed by the presence of the serum resistance-associated (SRA) gene (MacLean et al., 2004). The mechanisms behind this clinical variance are unclear but are likely associated with both host and pathogen factors. Elucidation of the roles of primate host immune factors in mediating outcomes of *T. b. rhodesiense* infection is essential for understanding the varied clinical outcomes in natural HAT cases but remains unexplored.

Cytokines are signaling molecules produced by immune cells and coordinate critical roles in the immune response, including responses to *T. brucei* infection (Kato et al., 2016). Cytokines regulate the proliferation, differentiation, and maturation of immune cells and facilitate communication between immune compartments. Consequently, their composition, magnitude, timing, and persistence modulate the intensity and duration of the response, which further determines protective and deleterious outcomes. In African trypanosomiasis, the early stage of infection triggers a pro-inflammatory type 1 immune response that is followed by an anti-inflammatory type 2 response at later stages (Baetselier et al., 2001; Musaya et al., 2015). Pro-inflammatory cytokines such as IFN-γ, TNF-α, and IL−1β mediate defense against the pathogen but are also associated with deleterious effects, such as more rapid progression and increased disease severity (MacLean et al., 2004). On the other hand, anti-inflammatory cytokines such as IL-4, IL-5, and IL-13 attenuate hyperinflammation and promote healing once the threat is neutralized or eliminated. Therefore, the balance between pro- and anti-inflammatory responses is important in controlling disease severity.

Cytokine dysregulation is reportedly associated with disease progression and severity in HAT (MacLean et al., 2004; Kato et al., 2015, 2016; Kamoto et al., 2021). These earlier studies were based on samples from clinical HAT patients and, for the most part, were limited in terms of accurate determination of disease onset, making the understanding of disease progression challenging. To address these issues, and specifically the absence of data from a primate model under controlled infection, we used the non-human primate (NHP) vervet monkey (*Chlorocebus aethiops*) model of HAT, which closely mimics human disease (Thuita et al., 2008), and a tsetse-mediated infection, as would be the case in the wild. We report on the association between cytokine production and the modulation of *T. b. rhodesiense* infection progression and disease outcomes.

## 2 Materials and methods

### 2.1 Ethics statement

Animal samples were collected as described in a previous study (Thuita et al., 2008) after approval by the Biotechnology Research Institute (BioRI) of the Kenya Agricultural and Livestock Research Organization (KALRO) Institutional Animal Care and Use Committee (IACUC), reference C/TR/4/490/1 (see **Text 1**).

### 2.2 Samples

**Figure 1:**
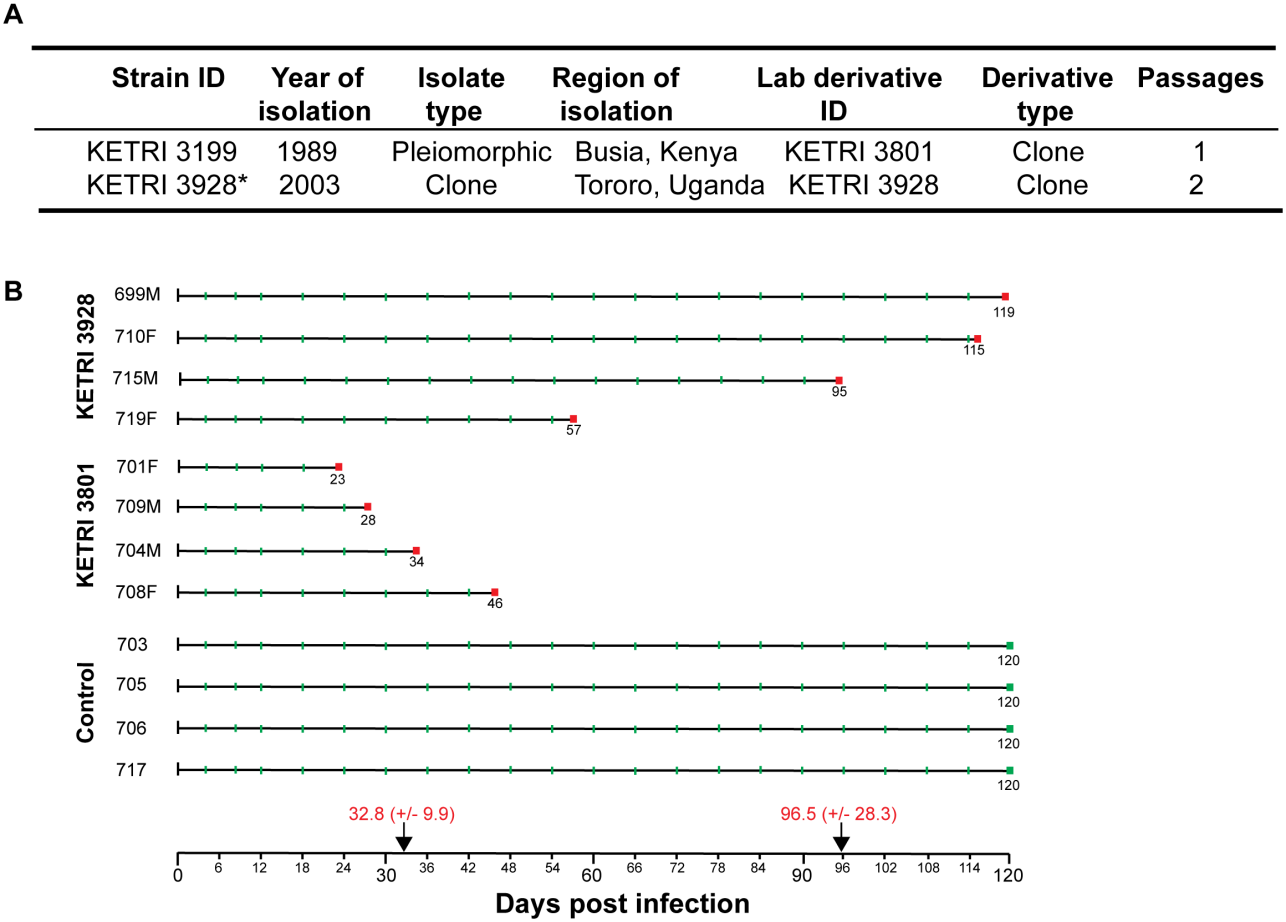
Strain biodata and sampling regime. **(A)** Biological and historical data for *T. b. rhodesiense* used in the study. **(B)** Eight vervet monkeys (*Chlorocebus aethiops*) were infected with the indicated strains of *T. b. rhodesiense*, while four served as uninfected controls. Blood and cerebrospinal fluid (CSF) samples were collected as indicated by green tick marks. Animals were euthanized at the times shown in red boxes following the emergence of clear late-stage symptoms (extremis). Mean survival times (± SD) are shown for the infected cohorts. F, female; M, male. These samples formed the resource for the study. *A cloned strain donated to the KALRO–BioRI laboratory by the National Livestock Resources Research Institute (NaLIRRI), Uganda.

Vervet monkey plasma and cerebrospinal fluid (CSF) samples were collected as described previously (Thuita et al., 2008), with modifications. Briefly, two groups of four animals each were infected with *T. b. rhodesiense* strains KETRI 3801 or KETRI 3928, which manifest as acute or chronic infections, respectively. A third group of four monkeys served as uninfected controls. Animals infected with KETRI 3801 were designated numbers 708 (F), 701 (F), 709 (M), and 704 (M). Those infected with strain KETRI 3928 were 710 (F), 719 (F), 699 (M), and 715 (M), while uninfected animals were 703 (F), 717 (F), 705 (M), and 706 (M). M and F represent male and female, respectively. Infection was mediated by a single bite from a tsetse fly confirmed to be infected with *T. b. rhodesiense* strains, as described by Thuita et al. (2008). Plasma samples were collected every 4 days until extremis, while CSF was collected from day 8 post-infection and every 4 days thereafter until extremis. An individual animal that, for 3 consecutive days, was either unable or reluctant to perch, had very low feed intake (<1/4 of the daily ration), and showed signs of advanced second-stage disease (e.g., somnolence) was considered to be in extremis and was thereafter humanely euthanized. Animals were first sedated using ketamine hydrochloride (10–15 mg/kg body weight, intramuscularly), after which a detailed clinical examination was carried out and 2 mL of venous (femoral) blood was sampled for a full hemogram. Euthanasia was performed using 20% pentobarbitone sodium (Euthatal, Rhône Mérieux). Strain biodata and the sampling regime are shown in **Figure 1**. Clinical data for the monkeys, including parasitemia (antilog_10_), CSF white blood cell (WBC) counts, packed cell volume (PCV), weight, temperature, survival time, and food consumption, were collected and analyzed. Plasma and CSF samples from three animals per cohort were used for cytokine analysis.

### 2.3 Immune factors

#### 2.3.1 Cytokine assays

Eight immune factors associated with varying African trypanosome infection outcomes in different HAT models (MacLean et al., 2001; Namangala et al., 2001; Sternberg et al., 2005; Kato et al., 2015, 2016) were considered, specifically IFN-γ, TGF-β1, TNF-α, IL-1β, IL-6, IL-10, IL-13, and IL-12. Also included was S100β, a serum biomarker for brain injury suggested as a marker for neuronal and blood–brain barrier (BBB) damage, making it a potential late-stage HAT biomarker (Rothermundt et al., 2003; Marchi et al., 2004; Bloomfield et al., 2007).

#### 2.3.2 Sample preparation and standards

Cytokine ELISA kits from U-Cytech Biosciences (Utrecht, Netherlands) were used to quantify monkey TNF-α, IFN-γ, IL-10, IL-6, IL-12, IL-13, and IL-1β in both plasma and CSF, as applied in previous studies (Laudenslager et al., 2006; Zhang et al., 2018; Willis et al., 2021). ABclonal Technology kits (Massachusetts, USA) were used to assay monkey TGF-β1 and S100β in CSF, as applied similarly elsewhere (Rothermundt et al., 2003; Marchi et al., 2004; Bloomfield et al., 2007). Samples (500 µL) were aliquoted into Eppendorf tubes to minimize freeze–thaw cycles. For U-Cytech Biosciences kits, 1/20 volume of cytokine stabilization buffer (CSB) was added to samples prior to subsequent dilutions. Additionally, 10 µL of balance solution was added to every 100 µL of diluted samples according to the ABclonal Technology protocol. To generate a standard curve, a seven-fold serial dilution of standards in dilution buffer was prepared, and the dilution buffer served as the blank.

#### 2.3.3 Cytokine assays

The U-Cytech Biosciences solid-phase sandwich ELISA protocol was followed according to the manufacturer’s instructions. Optical densities (ODs) of each well were read at 450 nm using a Stat Fax 3200 Microplate Reader (GMI, Minnesota, USA). Each sample was measured in triplicate. Incubation at 37 °C was performed using an MB100-4A microplate shaker incubator (MRC Ltd., Holon, Israel), while incubation at 4 °C was performed in an ALB fridge (ALB Service Pty Ltd., West Burleigh, Australia). Similarly, the ABclonal Technology protocol was applied according to the manufacturer’s instructions. Optical density was determined at 450 nm using a Stat Fax 3200 Microplate Reader (Awareness Technology Inc., Palm City, Florida, USA).

### 2.4 Statistical analyses

To monitor differences in cytokine levels over time between monkeys infected with *T. b. rhodesiense*, a generalized additive mixed model (GAMM) was used, with monkeys included in the model as a random effect. GAMM allowed modeling of highly non-linear profiles over time (Wood, 2017). Explanatory variables included strain (group), which entered the model parametrically as a fixed effect; time (days post-infection, dpi) as a nonparametrically smoothed function; and a time-by-strain smoothed term to allow each strain group to evolve differently over time. The uninfected group was used as the reference against which other groups were compared. GAMM was used to allow the inclusion of nonparametric smooth functions to model highly non-linear trends in the data. GAMMs measure nonlinearity using the effective degrees of freedom (edf) of the smoothing term. An edf equal to 1 implies a linear function, and the higher the edf, the more non-linear the function. GAMM was fitted using the gamm function in the mgcv package (Wood, 2004). This model was fitted separately for each cytokine using plasma data. Plasma data were analyzed for IFN-γ, TNF-α, IL-10, IL-6, IL-12, and IL-1β. Plasma IL-13 and CSF levels for all cytokines except TGF-1β and S100β, which exhibited no statistically significant alterations over the course of infection, were not subjected to statistical analyses.

Median survival times (and associated interquartile ranges, IQRs) were estimated using Kaplan–Meier curves (Kirkwood and Sterne, 2010), and the curves were compared across strains using the log-rank test. GAMM analyses were performed using R version 3.6.1 (R Core, 2019), and survival analysis was performed using Stata v15.1 (StataCorp, College Station, TX). All tests were performed at the 5% significance level.

## 3 Results

### 3.1 Pathogenicity of *T. b. rhodesiense* strains in vervet monkeys

**Figure 2:**
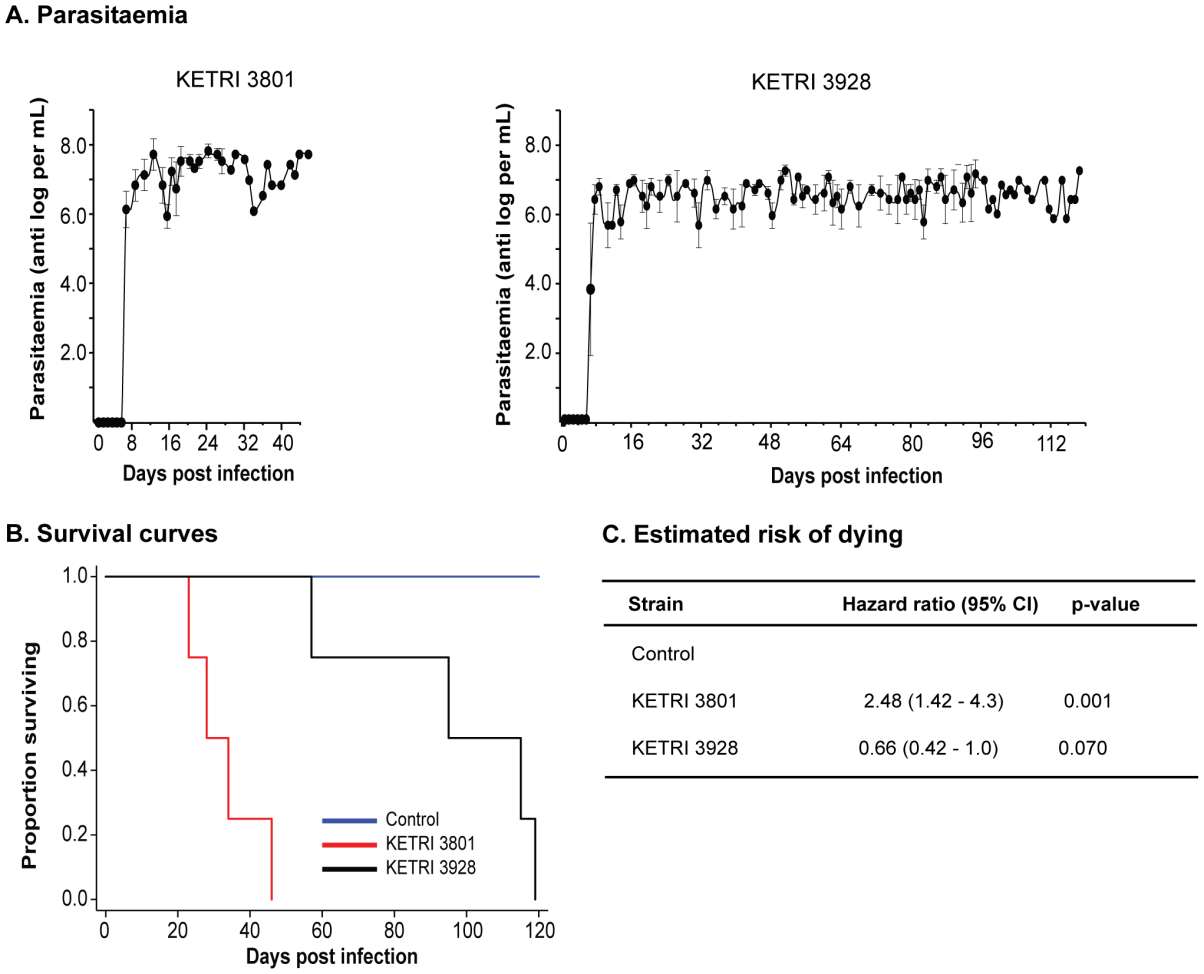
Parasitemia and survival of vervet monkey infected with different strains of *T. b. rhodesiense*. **(A)** The two strains, KETRI 3801 (left) and KETRI 3928 (right), exhibited first peak parasitemia above antilog 7.0 parasites/mL, followed by subsequent peaks, with a narrower range (6–8) observed in KETRI 3928 infection. **(B)** Kaplan–Meier survival curves for control, KETRI 3801-infected, and KETRI 3928-infected monkeys show that KETRI 3801 is the most virulent strain. Survival curves differed significantly across strains (log-rank p<0.001). **(C)** Estimated risk of death using a Cox proportional hazards model was higher in KETRI 3801 infection compared with KETRI 3928 infection.

Varying infection outcomes were observed following infection with the KETRI 3801 and KETRI 3928 strains and have been reported previously in both vervet monkey and mouse models (Thuita et al., 2008; Ndung’u et al., 2020; Limo et al., 2021). Here, a trypanosome chancre was observed in 1 (708) of the 4 monkeys infected with KETRI 3801 and in 2 (699 and 715) of the 4 monkeys infected with KETRI 3928. The median pre-patent period was 6.0 dpi (range: 6–6) and 6.3 dpi (range: 6–7) for KETRI 3801 and KETRI 3928 infections, respectively (**Table 1**). Following pre-patency, KETRI 3801 parasitemia rose to a peak of antilog 8.7 parasites/mL by 12 dpi before falling (**Table 1**); a second and third peak were observed before animals reached extremis (**Figures 1**, **2**). In contrast, KETRI 3928 parasitemia rose to a lower-magnitude peak of antilog 7.8 parasites/mL by 8 dpi (**Figure 2**). Parasitemia continued to fluctuate between antilog 6.1 and 7.8 parasites/mL throughout the infection (**Figure 2**). Overall, higher parasitemia was observed in monkeys infected with KETRI 3801 than in those infected with the KETRI 3928 strain (**Figure 2**).

**Table 1:** Changes to in clinical and parasitological parameters in vervet monkeys infected with different strains of *T. b. rhodesiense* (KETRI 3801 and KETRI 3928).

Clinical and parasitological parameters	Control (n = 4)	KETRI 3801 (n = 4)	KETRI 3928 (n = 4)
Proportion of monkeys with chancre	N/A	1/4	2/4
Pre-patent period (days)	N/A	6.0 (6–6)	6.3 (6–7)
Time to first peak parasitemia in dpi (parasite load/mL at first peak)	N/A	12 (antilog 8.7/mL)	8 (antilog 7.8/mL)
PCV (mean ± SEM %) at baseline (0 dpi)	49.3 ± 2.6	50.3 ± 5.2	51.6 ± 7.5
Percentage mean PCV change at day 12 dpi	-2.69	-13.27	-12.63
Percentage mean PCV change at extremis	-3.23	-44.90^46^	-59.60^115^
Time to first peak temperature (dpi)	N/A	12	8
Highest temperature increase (°C) in comparison to baseline (0 dpi)	0.68	1.80^12^	1.78^8^
Time to first detection of parasites in CSF (dpi)	N/A	[12][16][6][24]	[8][12][16][28]
Time to first detection of CSF white cell numbers > 5 cells/ul (dpi)	[-][-] [16][24]	[0][8][16][28]	[12][16][16][28]
Time to first detection of CSF white cell numbers > 5 cells/ul (dpi)	4.68 ± 0.77	4.50 ± 0.60	4.12 ± 0.60
Weight loss at extremis (Kg)	-0.6	-0.8	-1.2
Median (range) survival time (dpi)	120^‡^	28 (23–34)	95 (57–115)

“N/A” means not applicable since controls were uninfected. The number in superscript is the day at extremis for the last surviving animal in the cohort for PCV and the day of highest temperature increase in the course of infection. For the first dpi of detection of CSF parasites and white blood cell count (WBC) for individual animals per cohort, values are shown in square brackets; a “–” is used where none was detected. ^‡^ Represents the survival time of control animals and is censored data, since all were euthanized at the end of the study without attaining an extremis condition.

Animals infected with KETRI 3801 survived for shorter periods compared with those infected with KETRI 3928. The median survival time was 28 days (interquartile range [IQR] 23–34) for KETRI 3801, 95 days (IQR 57–115) for KETRI 3928, and 120 days (IQR 120–120) for controls (see **Table 1**; **Figures 1**, **2B**). Clinical monitoring of the control animals was terminated at 120 days without a requirement for them to attain the extremis condition.

Compared with KETRI 3928-infected monkeys, time to death was approximately one-third in KETRI 3801 infections (HR = 2.48, 95% CI: 1.42–4.30). However, the difference between KETRI 3928 infections and controls was not statistically significant (p=0.0704; HR=0.66, 95% CI: 0.42–1.00) (see **Figure 2C**). As in previous studies (Thuita et al., 2008; Limo et al., 2021), these data indicate that KETRI 3801 causes an acute infection compared with the chronic and milder KETRI 3928 infections.

**Figure 3:**
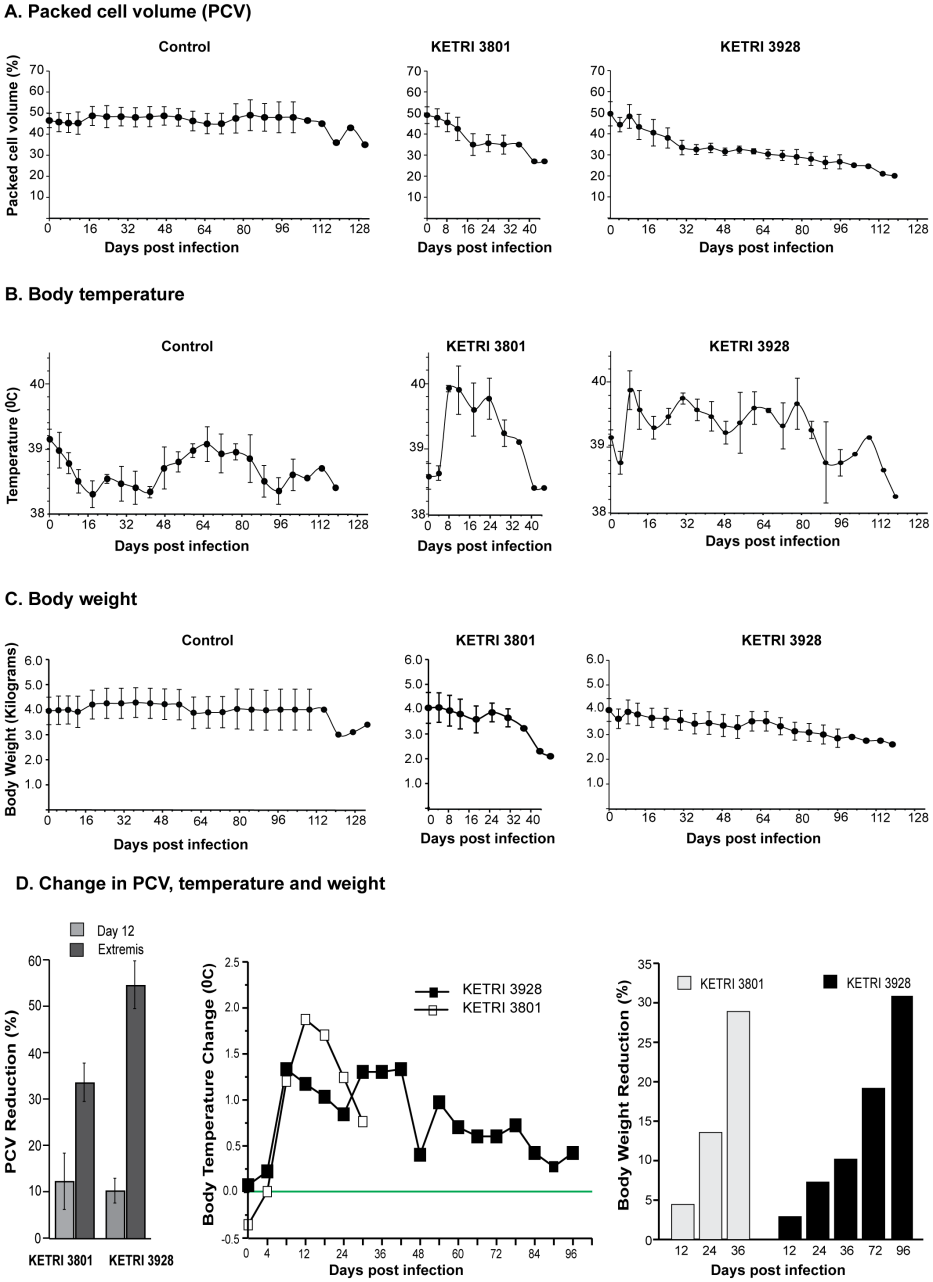
Impact of *T. b. rhodesiense* strains on vervet monkey. Effect on **(A)** packed cell volume (PCV), **(B)** body temperature, and **(C)** body weight of vervet monkeys infected with *T. b. rhodesiense* strains KETRI 3801 and KETRI 3928, which cause acute and chronic infections, respectively. Percent PCV and weight reductions at specific post-infection time points are shown in the **f**ar-left and far-right panels of **(D)**. Changes in body temperature at corresponding time points compared with controls are shown in the middle panel and are indicative of fever.

Reduced PCV level (percentage points) is an indicator of anemia. All infected animals exhibited progressive reductions in PCV during the course of infection, while control animals showed insignificant fluctuations (**Table 1** and **Figure 3A**). With reference to 0 dpi, the greatest reductions were observed at extremis, with an average reduction of 34% in KETRI 3801 and 55% in KETRI 3928 infections at termination of the experiment at 46 and 119 dpi, respectively (see **Figure 3D**, left panel). The rate of PCV reduction was higher in the KETRI 3801-infected cohort compared with the KETRI 3928 cohort (**Figure 3A**). An initial drastic reduction observed around 8–12 dpi coincided with the first peak parasitemia and was followed by a slight recovery, and thereafter a slow progressive reduction. Notably, the overall magnitude of PCV reduction correlated with survival time, with the highest reduction observed at extremis in monkeys infected with KETRI 3928, which survived the longest. This is indicative of the contribution of other factors, apart from PCV, to shorter survival times for KETRI 3801-infected animals. In addition, a higher rate of PCV reduction is likely associated with acute infections, as shown in **Figure 3A**.

Infected animals also had increased body temperatures compared with controls, an indication of fever (**Table 1** and **Figure 3B**). The highest average temperatures in KETRI 3801 (40.20 °C) and KETRI 3928 (40.03 °C) infections were observed at 12 and 8 dpi, respectively. These coincided with the first peak parasitemia. Body temperatures fluctuated throughout the study period within the ranges of 38.30–38.98 °C (Δ0.68), 38.40–40.20 °C (Δ1.80), and 38.25–40.03 °C (Δ 1.78) in controls, KETRI 3801, and KETRI 3928 infections, respectively. This suggests that greater temperature fluctuations are associated with infection. Overall, temperature increases in the monkeys were intermittent (**Figure 3D**, middle panel) and consistent with the well-known undulating fever associated with trypanosomiasis.

All infected animals registered weight loss, which increased with infection duration. Total body weight includes lean mass, fluids, and fat mass. Mean losses of 1.2 kg and 0.8 kg were observed in KETRI 3928 and KETRI 3801 infections, respectively (**Table 1**; **Figure 3C**). A higher rate of reduction in the KETRI 3801-infected cohort compared with the KETRI 3928-infected cohort was noted, with a loss of approximately 30% observed at extremis in both infections (**Figures 3C, D**, far-right panel). Longitudinally, a drastic and progressive decrease in body weight began following the first parasitemia peak in both infection cohorts. This was followed by a brief recovery in body weight, and thereafter a steady and drastic (for KETRI 3801 infections) or slow (for KETRI 3928 infections) decrease until extremis. With a standard feed ration and water provided *ad libitum* to all monkeys, infection-associated altered feeding and decreased feed intake, as shown in **Supplementary Figure S1**, could in part contribute to the observed weight loss. The varying rate of loss is strain-associated, with the highest total loss observed at extremis (see **Figures 3C, D**, far-right panel).

Together, PCV and body weight reductions were observed over the course of infection. Since water was provided *ad libitum* and all monkeys received a standard feed, weight reduction can be associated with infection. Further, infection-mediated fever was initially observed at the first peak parasitemia and persisted until extremis, with minor fluctuations. Comparatively, observations of PCV and weight loss, infection-mediated fever, median survival time, and risk of death indicate that KETRI 3801 can be considered acute, while KETRI 3928 infection is chronic. This is consistent with our work in the mouse model (Limo et al., 2021).

### 3.2 Cytokine changes with disease progression in the plasma

The outcome of an infection is the combined consequence of host and pathogen factors, interactions with the host environment (e.g., co-infection, nutritional status), and many other factors. An important host contribution is the immune response against the infectious agent(s), which may also contribute to immunopathology. Consequently, we sought to investigate immune factor alterations over the course of two differing infection outcomes, acute and chronic, to understand which, if any, immune factors may have roles in modulating the course of infection. Here, parasite-induced immune-mediated pathology due to varying alterations in quantities of pro-inflammatory cytokines (IL-1β, IL-6, TNF-α, IFN-γ), anti-inflammatory cytokines (TGF-β1 and IL-13), and those with both pro- and anti-inflammatory activity (IL-10 and IL-12) guided our selection. It is important to note that variation in pre-infection baseline cytokine levels due to factors such as age, sex, season, and gut microbiome (Mascarucci et al., 2001; McFarlane et al., 2011, 2012; Schirmer et al., 2016; Stone et al., 2021), among others, is normal and is responsible for the differences observed at day 0 post-infection.

**Figure 4:**
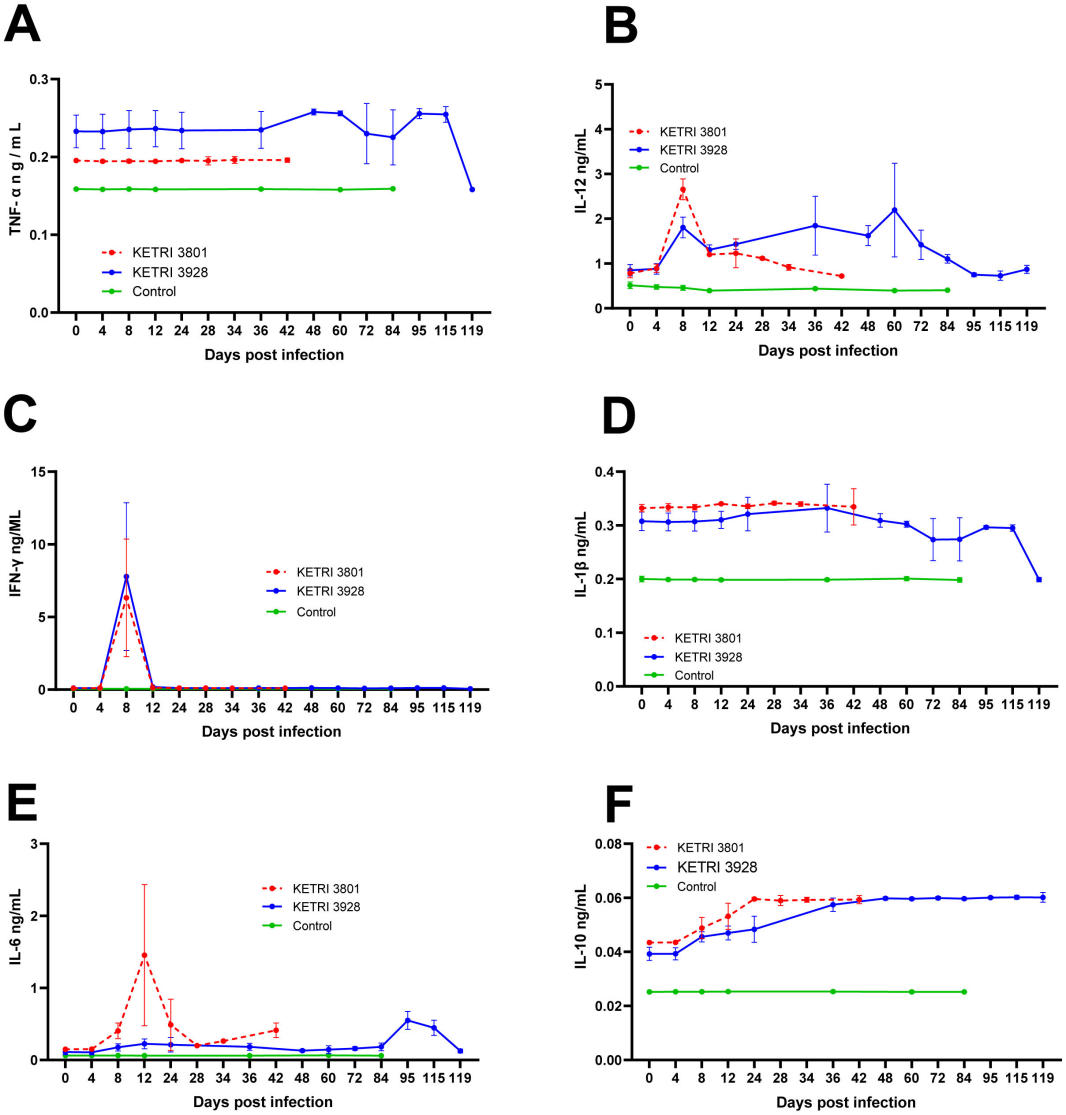
Plasma cytokine alterations in the course of *T. b. Rhodesiense* infection of vervet monkey. Graphs showing changes in various cytokines namely **(A)** TNF-α, **(B)** IL-12, **(C)** INF-γ, **(D)** IL-1β, **(E)** IL-6 and **(F)** IL-10 in the course of tsetse-mediated infection of vervet monkeys with two strains of *T. b. Rhodesiense* KETRI 3801 and KETRI 3928. The respective host immune factors were monitored till extremis for KETRI 3801 and termination of experiment for KETRI 3928 infection and control.

**Figure 5:**
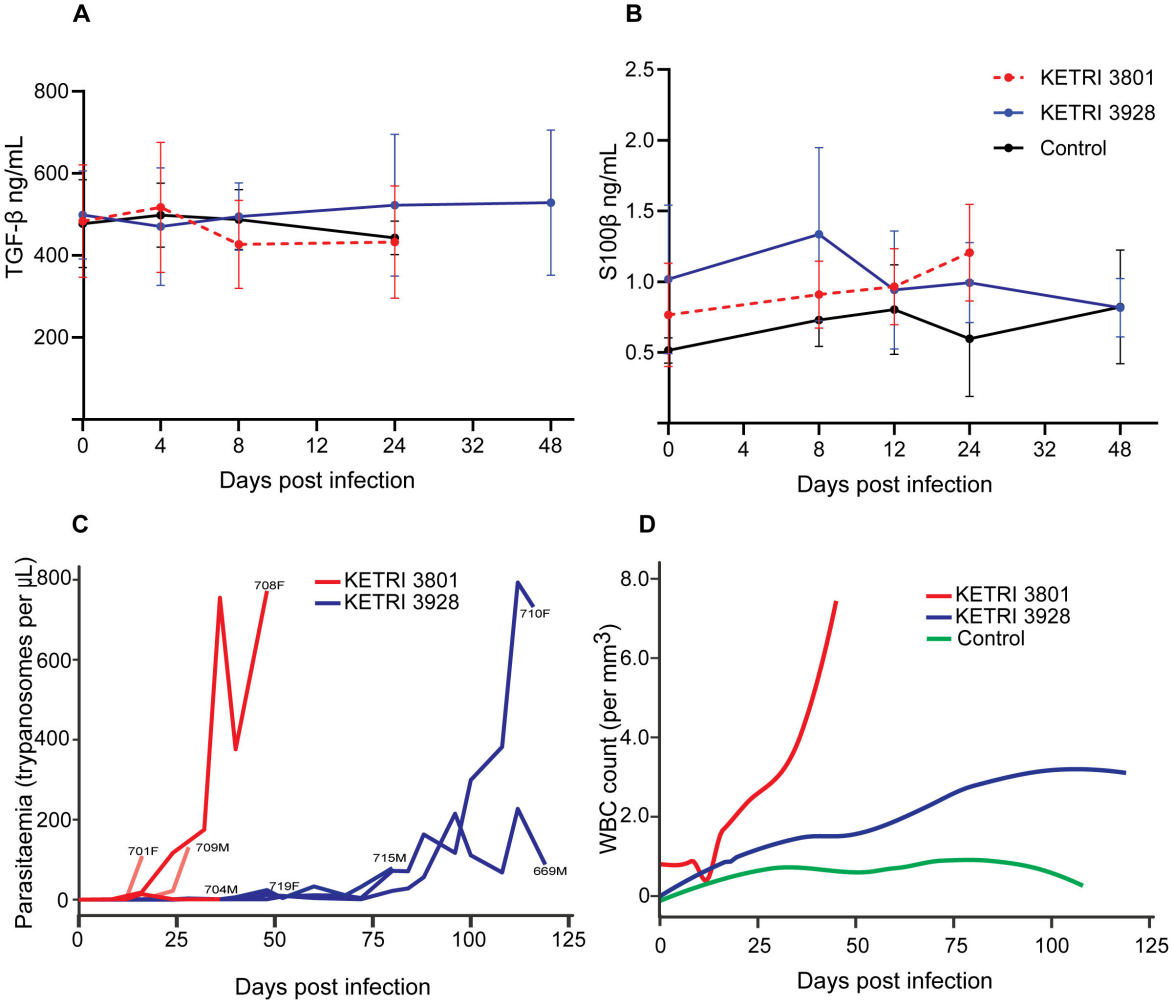
Cerebrospinal fluid (CSF) trypanosome invasion and associated immune factors levels in the course of infection of vervet monkeys with *T. b. rhodesiense* strains. **(A)** Profiles of TGF-1β and **(B)** S100β (ng/mL) during early stages of tsetse-mediated infection with *T. b. rhodesiense* strains KETRI 3801 and KETRI 3928. **(C)** Both parasite strains crossed the blood–brain barrier (BBB) into the CSF, causing stage two disease in all infected animals. **(D)** CNS invasion was accompanied by increased CSF white blood cell (WBC) counts. Graphs illustrate mean values for each cohort. Numbers in panel **(C)** denote individual animal codes.

Firstly, TNF-α is a broadly influential regulator of immunity and is produced by many immune cells. TNF-α plasma levels were elevated only in monkeys infected with the chronic strain, KETRI 3928 (**Figure 4A**). In controls and monkeys infected with the acute strain, TNF-α levels remained stable throughout the infection. A rise in TNF-α was observed after 48 dpi, when monkeys were confirmed to be in late-stage disease (**Table 1**; **Figures 5C, D**).

Secondly, IL-12 was monitored, as it plays a major role in T-cell differentiation, in particular Th1 and NK cells, as well as stimulating production of TNF-α. At baseline, levels of IL-12 ranged between 0.8 and 1.0 ng/mL in plasma. Following infection, IL-12 levels increased to a peak by 8 dpi in both groups of infected monkeys. In monkeys infected with KETRI 3801, IL-12 levels at 8 dpi were threefold higher than baseline. Thereafter, IL-12 levels declined but rose again to a second peak at 24 dpi (**Figure 4B**). In monkeys infected with KETRI 3928, the first peak of IL-12 was twice baseline and was observed at 8 dpi. Thereafter, IL-12 remained elevated above baseline, with another peak at 60 dpi. Terminally, IL-12 levels fell to baseline. The first peak of IL-12 was significantly greater in monkeys infected with KETRI 3801, and the profile differed compared with that of the KETRI 3928 strain (**Figure 4B**). As a bridge between pro- and anti-inflammatory responses, these varying levels indicate a crucial role in the immunopathologies associated with the two strains.

IFN-γ production is also influenced by TNF-α and is important for responses to a wide range of infections, inducing macrophage activation, and is produced by Th1 cells, among others. A single spike of IFN-γ was observed at 8 dpi in both groups of infected monkeys (**Figure 4C**), and no other peaks were observed thereafter. This suggests a similar effect in the infected cohorts. In controls, IFN-γ levels remained steady at baseline throughout the study duration.

We next analyzed soluble IL-1β levels, a cytokine involved in the inflammatory response and mainly produced by macrophages and monocytes. No notable peaks were observed in controls or monkeys infected with KETRI 3801. However, in monkeys infected with KETRI 3928, IL-1β levels in monkey 710 were elevated from 16 dpi, peaking at 40 dpi (**Supplementary Figure S2**), and showed wide variation between individuals in the cohort (**Figure 4D**). Thereafter, IL-1β levels subsided to levels similar to those of the other animals by 64 dpi.

IL-6 is associated with microbial infections, specifically through pathways mediated by Toll-like receptors. A first IL-6 peak was detected at 12 dpi in KETRI 3801-infected monkeys, while in the KETRI 3928-infected cohort this occurred terminally, at 95 dpi (**Figure 4E**). Comparatively, the increase was far more pronounced in KETRI 3801 infections than in KETRI 3928 infections, suggesting acute pathology.

IL-13 is an anti-inflammatory mediator, with effects on B cells resulting in increased IgE production. Here, plasma levels were very low and remained unchanged throughout the infection period (**Supplementary Figure S2**).

Finally, we considered IL-10, which has an anti-inflammatory function, downregulating Th1 cytokine synthesis and antigen presentation. An increase in IL-10 levels was observed after 4 dpi in both groups of infected monkeys, with those infected with KETRI 3801 showing higher levels (**Figure 4F**). At extremis, both infected cohorts had IL-10 levels that were nearly 1.5 times their baseline values. In both cohorts, this is indicative of attenuation of the pro-inflammatory response.

Taken together, the estimated effective degrees of freedom for cytokines indicate strong evidence for highly non-linear additive effects over time, except for TNF-α (KETRI 3801), IL-1β (KETRI 3801), and IL-6 (KETRI 3928) (**Supplementary Table S1**). In addition, plasma cytokine levels (TNF-α, IL-1β, IFN-γ, IL-6, IL-10, and IL-12) were, as expected, significantly higher in both groups of infected animals compared with controls (p < 0.05) (**Table 2**; **Figure 4**). Notably, levels of IL-6, IL-12, IL-10, and IFN-γ were significantly elevated in early-stage disease in both infected groups at 8–12 dpi, suggesting that these may be considered potential markers of early-stage disease. In addition, IL-6 and IL-12 levels were higher in acute compared with chronic infection and also exhibited differing profiles over time. These varying cytokine levels could contribute to the observed differential infection outcomes.

**Table 2:** Difference in average cytokine levels (ng/mL) between animals infected with two strains of *T. b. rhodesiense* compared with the control group, estimated using generalized additive mixed models.

Cytokine	Strain	Cytokine level difference	Std. error	t-value	*P*-value
TNF-α	Control	Reference	-	-	-
	KETRI 3801	0.036	0.008	4.4	<0.001
	KETRI 3928	0.073	0.008	9.7	<0.001
IFN- γ	Control	Reference	-	-	-
	KETRI 3801	1.216	0.377	3.2	0.001
	KETRI 3928	1.223	0.313	3.9	<0.001
IL-12	Control	Reference	-	-	-
	KETRI 3801	0.850	0.105	8.1	<0.001
	KETRI 3928	0.957	0.088	10.8	<0.001
IL-1 β	Control	Reference	-	-	-
	KETRI 3801	0.134	0.010	13.4	<0.001
	KETRI 3928	0.095	0.009	10.4	<0.001
IL-6	Control	Reference	-	-	-
	KETRI 3801	0.436	0.061	7.2	<0.001
	KETRI 3928	0.121	0.051	2.4	0.018
IL-10	Control	Reference	-	-	-
	KETRI 3801	0.026	0.002	11.2	<0.001
	KETRI 3928	0.026	0.002	12.5	<0.001

A positive value implies that cytokine levels in infected animals are higher than those in controls.

### 3.3 Cytokine changes with disease progression in the cerebrospinal fluid

To stage sleeping sickness, either the presence of parasites and/or >5 WBC/µL in the cerebrospinal fluid (CSF) is considered an indicator of late-stage (stage two) disease (WHO, 1998). Parasites were detected in the CSF between 12–24 dpi and 8–28 dpi in KETRI 3801- and KETRI 3928-infected animals, respectively, across all infected cohorts (**Table 1**; **Figure 5C**). Notably, earlier central nervous system (CNS) invasion cannot be excluded due to the intervals of lumbar puncture used for the determination of parasites and WBC. Similarly, CSF WBC counts >5 cells/µL were noted between 0–28 dpi and 12–28 dpi in KETRI 3801- and KETRI 3928-infected animals, respectively. For the control cohort, WBC counts >0.5 cells/µL were detected in two animals at days 16 and 24 (**Figure 5D** and **S3**; **Table 1**). Overall, there was a more rapid increase in CSF parasite and WBC levels in KETRI 3801 compared with KETRI 3928 infections (**Figures 5C, D**), which is again indicative of more rapid parasite replication and/or invasion, progression, and severity of late-stage disease in KETRI 3801 infections compared with KETRI 3928 infections.

In the CSF, levels of six cytokines were extremely low, except for IL-12 (in monkeys 715 and 709; **Supplementary Figure S2**), TGF-1β and S100β. A limitation of this analysis is that marker levels were low and often at or below detectable limits (Tarrant, 2010). TGF-1β has multiple roles in inflammation, mainly acting to suppress B-cell and macrophage activity, as well as promoting differentiation of CD4 T cells. A variable TGF-1β response was observed within the monkey cohort. In monkeys infected with KETRI 3801, two animals (708 and 704) showed an increasing trend relative to baseline, with peaks at 4 dpi and 24 dpi (**Figure 5** and **S2**). In monkeys infected with KETRI 3928, levels in monkey 699 were elevated throughout the infection (**Supplementary Figure S2**), with variable responses observed in the remaining animals.

S100β is secreted by mature astrocytes and is a marker of CNS injury and blood–brain barrier permeability. Wide variations in S100β levels were observed. In KETRI 3801-infected monkeys, two individuals (704 and 708) showed increased levels during infection. In contrast, a variable response with no clear trend was observed in KETRI 3928-infected monkeys (**Supplementary Figure S2**).

In summary, the majority of CSF immune factors assayed remained unchanged throughout the course of infection, except for IL-12 (in two animals), TGF-1β and S100β. Furthermore, higher CSF parasite and WBC counts were observed earlier in second-stage disease in KETRI 3801 (acute) compared with KETRI 3928 (chronic) infections. The full dataset is provided as **Dataset 1** in the **Supplementary Information**.

## 4 Discussion

African trypanosomes have multiple and elaborate strategies to evade mammalian host defenses, permitting long-term survival and an increased transmission window. Among these is antigenic variation, a periodic switch of the major surface coat protein, variable surface glycoprotein (VSG), to avoid detection and clearance by host antibodies. VSG also forms a protective layer for invariant surface molecules, including invariant surface glycoprotein 65 (ISG65), which limits complement-mediated killing (MacLeod et al., 2022; Lorentzen et al., 2023; Sülzen et al., 2023), and ISG75, which binds Fc regions of immunoglobulins (Mikkelsen et al., 2024). Further, surface hydrodynamic flow on motile parasites drags antibody–surface protein complexes to the flagellar pocket (Engstler et al., 2007). These complexes are subsequently internalized, antibodies degraded, and the surface proteins, including VSGs, recycled back to the surface (Engstler et al., 2004; Koumandou et al., 2008). This limits antibody-mediated clearance and, with membrane trafficking upregulated in bloodstream-stage parasites (Koumandou et al., 2008), offers considerable defensive protection.

Additional resistance mechanisms operate in human-infective trypanosomes through the expression of serum resistance-associated (SRA) protein and the *T. b. gambiense*-specific glycoprotein (TgsGP), which neutralize human serum trypanolytic factor (TLF) (Xong et al., 1998; Capewell et al., 2013). While these defense strategies are well described, our understanding of trypanosome-directed suppression and/or alteration of host immune responses to favor parasite survival remains limited. In addition, differential immune responses, as determined by trypanosome species and strain, host species, and mode of infection (Wei et al., 2011), influence infection outcomes despite the common presence of these immune evasion systems. Using the non-human primate (NHP) model of HAT, we interrogated the evolution of clinical and immune responses in vervet monkeys infected with two strains of *T. b. rhodesiense* responsible for differing infection outcomes: acute (KETRI 3801) and chronic (KETRI 3928) sleeping sickness.

Tsetse-mediated infection via a single blood meal bite (Thuita et al., 2008) mimics natural infection and was accompanied by the presence of a trypanosomal chancre at the bite site in some animals. The chancre results from a localized inflammatory response to inoculated parasites (McGovern et al., 1995; Sekhar et al., 2014), tsetse salivary components (Caljon et al., 2006; Alfituri et al., 2021), and possibly tissue laceration caused by probing for hemorrhagic pool feeding. It represents the host’s first attempt to prevent and contain initial parasite colonization. However, a chancre is not observed following all infectious bites and is suggested to vary depending on both infecting species and host (Urech et al., 2011; Crilly and Mugnier, 2021). The molecular basis underpinning this variability remains unknown and may not depend on parasite virulence alone but rather reflect the effects of the tsetse bite itself.

Thereafter, we observed higher primary peak parasitemia, replication rate, and overall parasitemia in strain KETRI 3801 compared with KETRI 3928 (see **Figure 2A**). A similar trend has been observed in the mouse model (Limo et al., 2021), where differences in strain-dependent factors affecting replication (proliferation and differentiation) and host-mediated clearance likely play a role. In addition, lower parasitemia in KETRI 3928 infections is characteristic of chronic trypanosome infections, as previously reported by Turner et al. (1995). It is possible that KETRI 3928 maintains sublethal parasitemia by balancing host clearance with differentiation into non-dividing stumpy forms, which are more resistant to complement-mediated clearance than slender forms (McLintock et al., 1993; Engstler et al., 2007). This may limit adverse effects on the host, resulting in longer host survival and an extended transmission window. Observations by Murray and Morrison (1978), indicating that higher parasitemia is associated with increased pathogenesis, partly explain the adverse pathology observed in KETRI 3801 infections.

We observed acute (KETRI 3801) and chronic (KETRI 3928) infection outcomes, as inferred from their respective impacts on disease progression (anemia, loss of body weight, and survival probability) and overall survival (**Table 1**; **Figures 1**–**3**). These outcomes are most likely strain-dependent. First, short (28 days) and long (95 days) (**Figures 1B**, **2B**, and **Table 1**) survival is observed, respectively, in KETRI 3801 and KETRI 3928 infections of both vervet monkeys and mice (Limo et al., 2021). In addition, the risk of death was threefold higher in KETRI 3801 infections compared with KETRI 3928 infections (**Figure 2C**). Second, hosts within each cohort consistently exhibited similar outcomes, indicative of strain-associated effects. If this were not the case, significantly greater variability in measured parameters would be expected within cohorts. We therefore suggest that immunopathologies differentially manifested between the KETRI 3801- and KETRI 3928-infected cohorts are a consequence of strain-directed differential immune responses. This implies that immune cells and factors (e.g., cytokines, chemokines, growth factors, and signaling molecules), together with their quantities, dynamics, and/or localization, determine the evolution of immune responses and immunopathology and, ultimately, infection outcome.

Anemia is a common feature of African trypanosomiasis and results from immunopathology-mediated erythrophagocytosis and/or reduced erythropoiesis. Persistent early type 1 pro-inflammatory responses are associated with acute anemia, while subsequent IL-10-mediated resolution into a type 2 anti-inflammatory response manifests as chronic anemia. Here, we infer that strain-directed immunomodulation contributes to differences in anemia severity (see **Figure 3A**), with pro-inflammatory cytokines IL-1β, IL-6, IFN-γ, and TNF-α implicated. For example, as observed in humans, rhesus macaques, and murine models, IL-6—which was sixfold higher in KETRI 3801 than in KETRI 3928 infections at 12 dpi (**Figure 4E**)—can, in a reversible and dose-dependent manner (van Gameren et al., 1994; Winton et al., 1994; Neves et al., 2021), reduce serum iron levels (Neves et al., 2021), thereby suppressing erythropoiesis. Similarly, IFN-γ (**Figure 4C**) may contribute by inhibiting erythroblast proliferation and differentiation, suppressing erythropoiesis, as observed elsewhere (Felli et al., 2005; Cnops et al., 2015). In contrast, IL-12, which remained moderately elevated throughout KETRI 3928 infection but decreased toward extremis (**Figure 4B**), is a dose-dependent stimulator of erythropoiesis (Dybedal et al., 1995) and is protective against anemia (Zhang et al., 2010). Its temporal profile during infection may permit a gradual loss of protection, resulting in chronic anemia. In summary, we infer that strain-directed IL-6, IFN-γ, and IL-12 responses likely contribute to the acute and chronic anemia observed in infected monkeys.

Food and water were provided *ad libitum*, yet weight loss was observed and was greater in acute compared with chronic infections (**Figure 3C, D**; **Table 1**). This loss is most likely infection-induced and strain-dependent. Two reasons could account for this. First, infection-induced hypophagia (**Supplementary Figure S1**), a feature also observed in murine models of African trypanosomiasis (Limo et al., 2021; Trindade et al., 2016), worm infections (Worthington et al., 2013), and loss of appetite in infected humans. Hypophagia could be a consequence of satiety signals from feed control centers of the brain, mediated directly or indirectly by immune factors and/or hormones such as leptin (Ahima and Antwi, 2008; Maurya et al., 2018). Second, immune-directed reduction of components of body weight (i.e., fluid, lean, and fat mass), as recently observed in murine models of African trypanosomiasis (MaChado et al., 2023; Redford et al., 2023), could be contributory. Inference from *in vitro* and mouse model studies suggests that hypophagia and immune-mediated reductions in lean and fat mass can explain the weight loss observed in our vervet monkey model of HAT.

Fever is part of the acute-phase response and has a protective role (LeGrand and Day, 2016). Febrile temperature acts as a stressor, harming replicating pathogens (LeGrand and Alcock, 2012), increasing their susceptibility to destruction, and stimulating both innate and adaptive immune responses (Evans et al., 2015). This protective role could explain reductions in parasitemia following peak febrile temperatures observed here. In addition, characteristic intermittent infection-associated fevers point to the host’s ability to control intensity and timing, indicative of a host-driven response rather than a direct pathogen-driven process. The central endogenous pyrogenic mediator is IL-6, augmented by other mediators such as IL-1, TNF-α, cyclooxygenase-2 (COX-2), and prostaglandin E_2_ (PGE2) (Sundgren-Andersson et al., 1998; Cartmell et al., 2000; Li et al., 2003; Nilsberth et al., 2009). Also required are pathogen factors that act as exogenous pyrogens, whose detection by resident innate immune cells elicits IL-1 and IL-6 production and activates PGE2 synthesis (Martínez-Colón and Moore, 2018). Trypanosome glycosylphosphatidylinositol (GPI) is a prime candidate exogenous pyrogen and has been shown to elicit innate cytokine production, including IL-6 (TaChado and Schofield, 1994; Magez et al., 1998; Stijlemans et al., 2007). The higher levels of IL-6 observed in infected cohorts (**Table 2**; **Figure 4E**) could explain increases in core body temperature, while differences in febrile responses between acute and chronic infections may reflect strain-related variation in fever mediators. For example, differential IL-6 activation of COX-2 in endothelial cells of hypothalamic microvessels (Yamagata et al., 2001; Rummel et al., 2006), and consequently cerebral PGE2 levels, acting in a dose-dependent manner, could be involved. It is plausible that pyrogenic cytokines and pathogen-derived factors together contribute to fever in this NHP model of HAT.

Crucial in determining infection outcomes are immune factors linking innate and adaptive immune responses, as they regulate both protective and deleterious aspects of inflammation. Here, key crossroad mediator candidates are IL-12 and IL-10 and their downstream factors. For example, the pro- and anti-inflammatory cytokine IL-6 (Scheller et al., 2011), whose secretion is influenced by IL-12 (Cahill and Rogers, 2008; Wu et al., 2017; Kak et al., 2018), may be important in the resolution of immune responses and the outcomes observed here. Elevated IL-6 levels, as seen in acute KETRI 3801 infection, are associated with highly virulent strains in other infections (Ng et al., 2009; Velazquez-Salinas et al., 2018) and are predictive of disease severity, as reported in bovine trypanosomiasis (Kuriakose et al., 2016). IL-6 levels would differentially modulate pro-inflammatory responses mediated by IFN-γ, whose levels were similar in both cohorts. Similarly, IL-10 promotes pathogen-eliminating pro-inflammatory responses but also suppresses or attenuates hyperinflammation to limit immune-mediated tissue damage. As shown in **Figure 4F**, we observed steady upregulation of IL-10, consistent with findings by Ngotho et al. (2006), followed by maintenance at higher levels. The early upregulation after 4 dpi (as also observed for IL-6, IL-12, and IFN-γ; **Figure 4**) may indicate initial efforts to control hyperinflammation and potential tissue damage while maintaining protective immunity. The slow progressive increase may permit rapid activation of innate and adaptive responses for effective pathogen control. Thereafter, sustained elevated levels may become immunosuppressive, favoring host tolerance and prolonged pathogen survival in chronic infections. Similar responses have been proposed in various infections, including *Toxoplasma gondii* (Wilson et al., 2005), leishmaniasis (Anderson et al., 2008), and viral infections (Ribeiro et al., 2021), and could explain the lower but steady parasitemia observed in KETRI 3928 infections. Therefore, differential expression of IL-12 and IL-10, and their downstream mediators (e.g., IL-6), may play a pivotal role in crosstalk between innate and adaptive immunity, indicative of varying immunomodulation and immunopathology that manifest as acute and chronic infection outcomes.

Positive detection of parasites in blood is accompanied by disease staging that involves a painful and invasive lumbar puncture to determine CSF parasites and/or WBC counts. Here, while the timing of CNS invasion (between weeks 6 to 21 dpi; **Table 1**) did not differ between infections, we observed higher CSF parasitemia early in KETRI 3801 (acute) compared with KETRI 3928 (chronic) infections (**Figure 5C**). CSF is a unique and hostile microenvironment (Pentreath, 1999), and reasons for variation in parasitemia could include: (i) retention of replication rates between bloodstream form (BSF) and CSF forms, corroborated by observations in mice (Ndung’u et al., 2008); (ii) variable parasite survival in CSF; or (iii) varying bidirectional transmigration if invasion is, in part, influenced by BSF levels and pathogen-directed immunomodulation, as suggested by others (Mogk et al., 2014; Laperchia et al., 2016). In addition, increases in WBC counts following parasite CNS invasion and with disease progression suggest parasite-induced recruitment, as observed by Frevert and colleagues (Frevert et al., 2012) in the murine model. Considering individual infected animals within each cohort and their survival times, a combination of additional factors together with CSF parasitemia is likely implicated in late-stage fatality. Our preliminary observations using these strains in a vervet monkey model of HAT could be valuable for interrogating mechanisms of CNS invasion and associated impairments, as well as improving understanding of the timing and evolution from early- to late-stage disease, given that neurological characteristics have been observed in early-stage patients (MacLean et al., 2010).

Differential immune responses, and hence variation in disease progression and infection outcomes, are likely influenced in part by pathogen factors. Here, trypanosome-derived factors at the host–pathogen interface that modulate host immune responses are candidates. Examples include adenylyl cyclase (AC) (Salmon et al., 2012), soluble VSGs (Coller et al., 2003), kinesin heavy chain (KHC) (De Muylder et al., 2013), stumpy induction factor (SIF) (Bakhiet et al., 1993), and parasite metabolism and secreted metabolites (Holzmuller et al., 2008, 2018; Diskin et al., 2021; Levy et al., 2021), among others. To permit varying immunomodulation, strain-specific differences in trypanosome gene expression and, consequently, interactions with the host are likely. For example, differences in parasitemia levels could, in part, reflect varying populations of stumpy forms due to different levels of SIF. SIF could consequently be indirectly responsible for variation in immune responses through IFN-γ, whose biosynthesis it has been shown to trigger (Bakhiet et al., 1993). In addition, differential proteomic and/or secretomic profiles associated with virulence and pathogenicity have been observed in strains of *T. b. gambiense* (Holzmuller et al., 2008), *T. vivax* (Ramirez-Barrios et al., 2019), and *T. congolense* (Grébaut et al., 2009). Our preliminary studies have shown differential transcriptomes between the two *T. b. rhodesiense* strains, indicative of potentially distinct interactions with the vervet monkey host and, hence, differing infection outcomes.

In summary, we demonstrate distinct immune and clinical impacts of two *T. b. rhodesiense* strains, KETRI 3801 and KETRI 3928, responsible for acute and chronic infection outcomes, respectively. These differences are inferred from anemia, weight loss, risk of death, and survival time. We suggest a possible association between strain-directed and host-dependent immunomodulation as the basis for the observed differences in infection outcomes. Further studies will be required to define the individual roles of immune components, including immune cells, cytokines, chemokines, and other immunomodulatory agents, and to integrate these into their complex interaction networks. Such insights will be important for understanding HAT progression, identifying potential staging biomarkers, and improving clinical management. In the context of vector-transmitted pathogens, maladaptation of strains causing acute infections—marked by short host survival and a narrow transmission window—may occur, whereas strains responsible for chronic infections may exhibit higher transmission potential in the wild.

## Data Availability

The original contributions presented in the study are included in the article/**Supplementary Material**. Further inquiries can be directed to the corresponding author.

## Supplementary material

Supplementary Text 1 Ethical approval for animal use referenced C/TR/4/490/1.

Supplementary Table 1 Estimated degrees of freedom showing trends of plasma cytokines with disease progression.

Supplementary Figure 1 Hypophagia observed in vervet monkey infected with KETRI 3801 and KETRI 3928 strains of *T. b. rhodesiense*.

Supplementary Figure 2 Plasma and cerebrospinal fluid (CSF) cytokine and S100β changes in the course of *T. b. rhodesiense* infection in vervet monkeys.

Supplementary Figure 3 Cerebrospinal fluid (CSF) white blood cell (WBC) count.

Supplementary Data Sheet 1 Clinical, parasitological and immunological data of vervet monkeys infected with two strains of *T. b. rhodesiense* over the course of infection.
