# Supplementary material for: Roles of cytokines in modulating Trypanosoma brucei rhodesiense infection outcomes in vervet monkeys
Source: Front Parasitol. 2026 Jan 12;4:1725651. doi: 10.3389/fpara.2025.1725651 (PMC12833445; doi:10.3389/fpara.2025.1725651)
Supplement: Supplementary Text 1 — Ethical approval for animal use referenced C/TR/4/490/1. [file DataSheet1.zip › Table S1.DOCX]

**Table S1**. Estimated degrees of freedom showing trends of plasma cytokines with disease progression.

| **Cytokine** | **Strain** | **E.df** | **Ref.df** | **F-value** | ***P*-value** |
| --- | --- | --- | --- | --- | --- |
| TNF-α | KETRI 3801 | 0 | 7 | 0 | 0.999 |
|  | KETRI 3928 | 5.498 | 8 | 3.229 | <0.001 |
| IFN- γ | KETRI 3801 | 2.567 | 8 | 1.375 | 0.004 |
|  | KETRI 3928 | 4.970 | 8 | 2.548 | <0.001 |
| IL-12 | KETRI 3801 | 3.313 | 8 | 4.821 | <0.001 |
|  | KETRI 3928 | 6.436 | 8 | 8.660 | <0.001 |
| IL-1 β | KETRI 3801 | 0 | 8 | 0 | 0.554 |
|  | KETRI 3928 | 7.307 | 8 | 27.1 | <0.001 |
| IL-6 | KETRI 3801 | 3.591 | 8 | 9.089 | <0.001 |
|  | KETRI 3928 | 0 | 8 | 0 | 0.385 |
| IL-10 | KETRI 3801 | 3.724 | 8 | 55.17 | <0.001 |
|  | KETRI 3928 | 7.589 | 8 | 155.7 | <0.001 |

E.df is effective degrees of freedom, and measures nonlinearity, with a value of 0 implying a linear function while a greater value indicates nonlinearity. Ref.df is the reference degrees of freedom and is used in hypothesis testing and p value calculations of the smooth terms.
