# Supplementary material for: Roles of cytokines in modulating Trypanosoma brucei rhodesiense infection outcomes in vervet monkeys
Source: Front Parasitol. 2026 Jan 12;4:1725651. doi: 10.3389/fpara.2025.1725651 (PMC12833445; doi:10.3389/fpara.2025.1725651)
Supplement: Supplementary Text 1 — Ethical approval for animal use referenced C/TR/4/490/1. [file DataSheet1.zip › Text 1.PDF]

(1)

KENYA AGRICULTURAL RESEARCH INSTITUTE

Trypanosomiasis Research Centre, MUGUGA

P.O. BOX 362, KIKUYU, 00902, TEL: 020-2700604, 2700654, FAX: 066-32397

KIKUYU

Email:Centre@ Kari-trc.org, trccd@kari-trc.org

Our Ref: C/TR/4/490/1 **13/3/ 2015**

Drs John Thuita and Dan Masiga,
Principal Investigators (PIs).

Dear John and Dan

RE: IACUC Approval

The Institutional Animal Care and Use Committee (IACUC) of the Trypansomiasis Research Centre-Kenya Agricultural Research Institute (TRC-KARI) has reviewed your proposal entitled "Expression Profiling of African trypanosomes in Mouse and Primate hosts". The committee notes your study aims to profile human infective African trypanosomes with the objective of identifying potential biomarkers for diagnosis and disease staging. This is an important study topic with potential benefits for the control of humanAfrican rypanosomaisis (HAT). The study methods addressing the *in vivo* component of your study are appropriate and are consistent with IACUC recommendations as well as National (KVA) standards of animal welfare. The committee has therefore resolved to support your study. Note that the committee will hold you and your co-investigators personally responsible to ensure that appropriate standards of animal welfare are observed at all times. The committee may make impromptu visits to ensure compliance with its regulations.

Yours Sincerely

Dr Carolius Oidho
For Chairman, Institutional Animal Care and Use Committee (IACUC)
TRC-KARI
