# Supplementary figures and images for: Roles of cytokines in modulating Trypanosoma brucei rhodesiense infection outcomes in vervet monkeys
Source: Front Parasitol. 2026 Jan 12;4:1725651. doi: 10.3389/fpara.2025.1725651 (PMC12833445; doi:10.3389/fpara.2025.1725651)

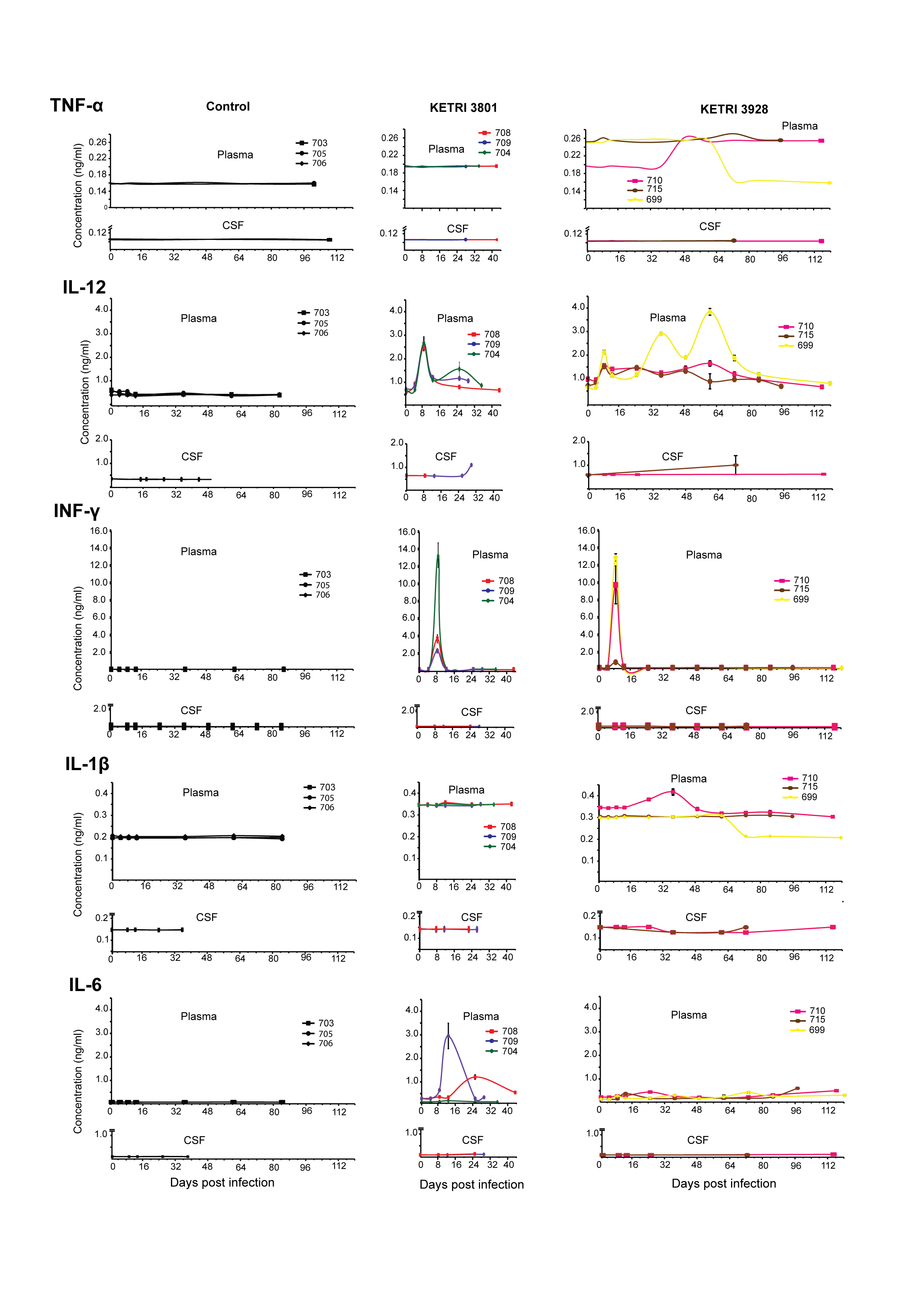

Supplement: Supplementary Text 1 — Ethical approval for animal use referenced C/TR/4/490/1. [file DataSheet1.zip › Figure S2A.TIF]

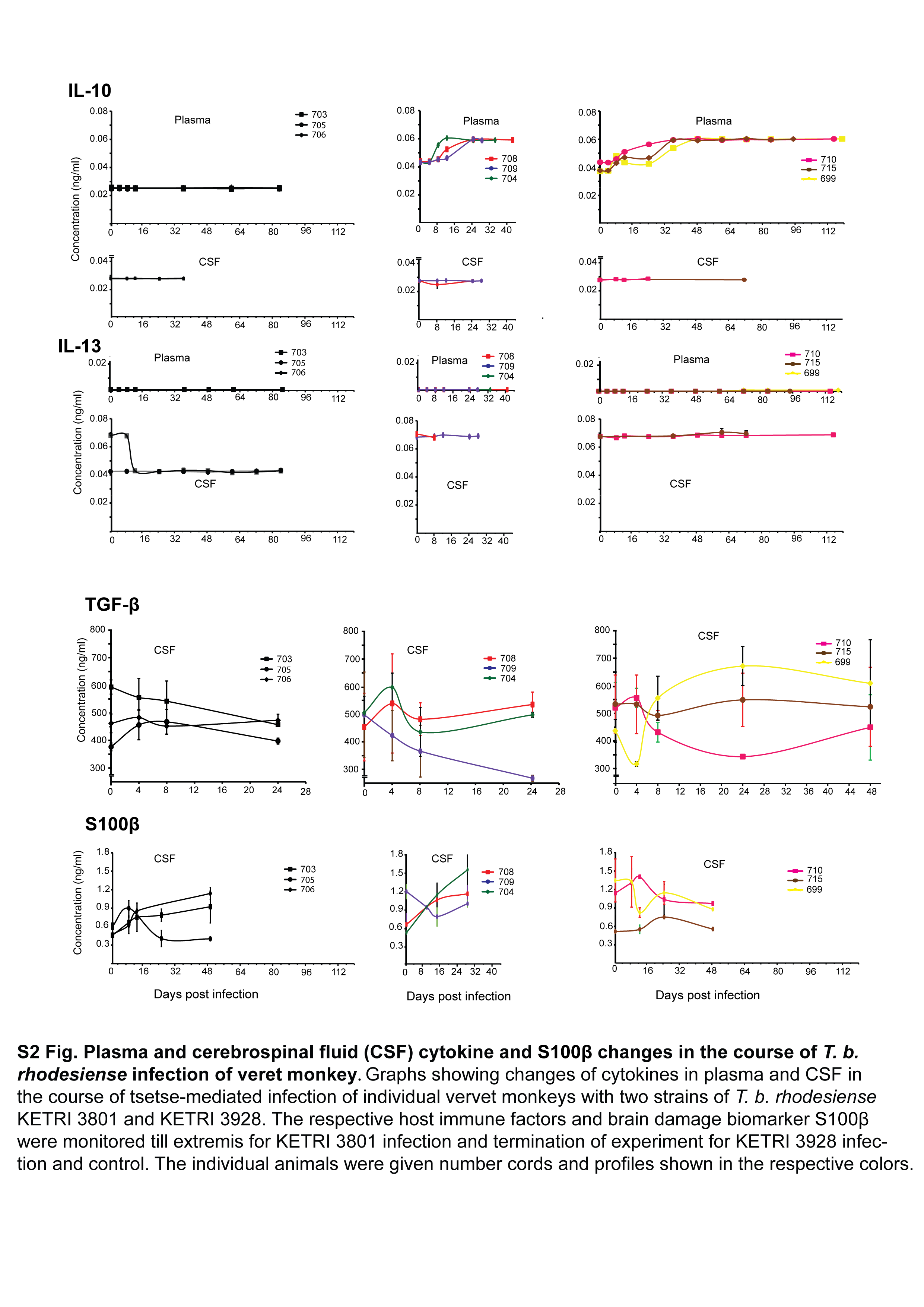

Supplement: Supplementary Text 1 — Ethical approval for animal use referenced C/TR/4/490/1. [file DataSheet1.zip › Figure S2B.TIF]

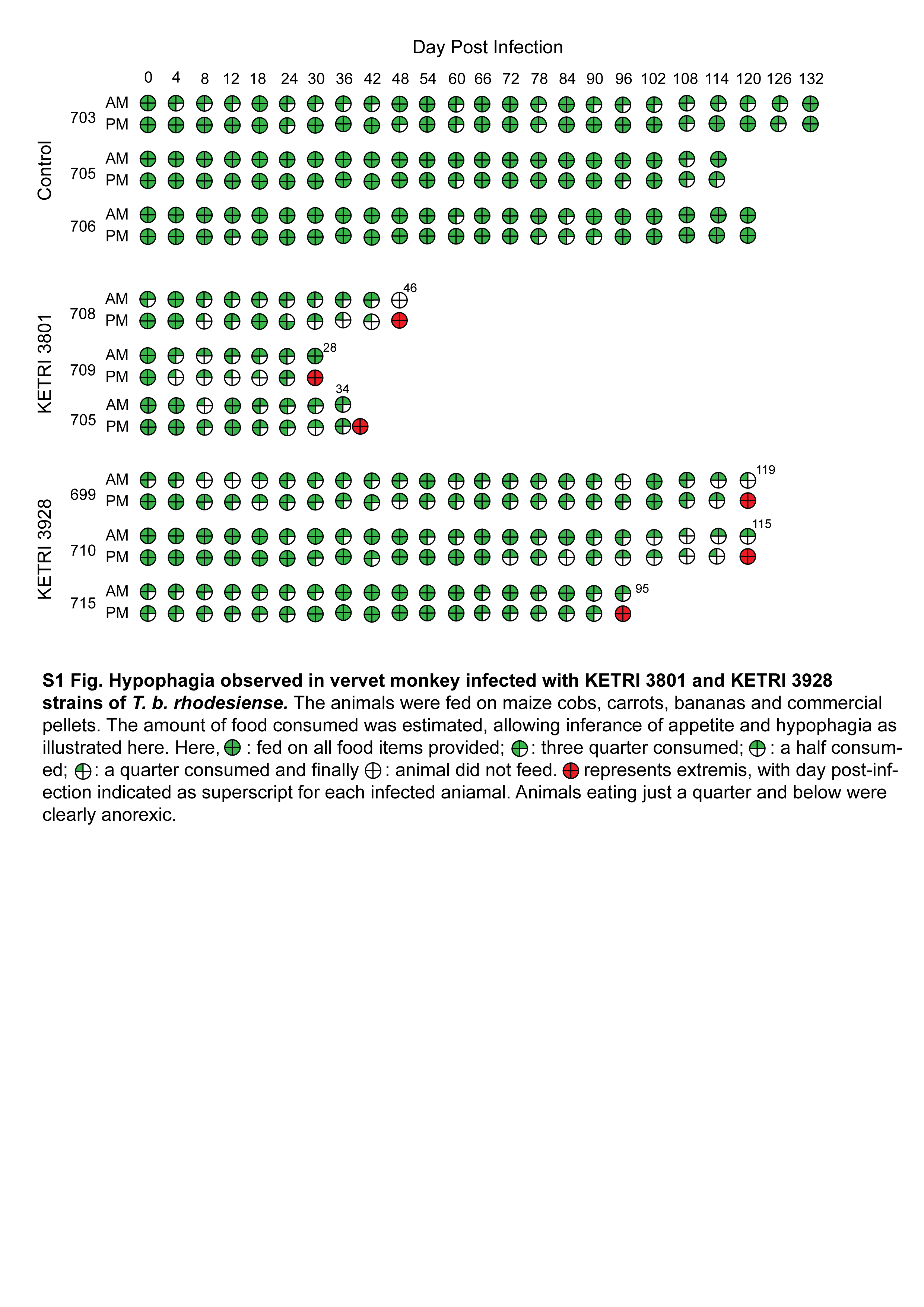

Supplement: Supplementary Text 1 — Ethical approval for animal use referenced C/TR/4/490/1. [file DataSheet1.zip › Figure S1.TIF]

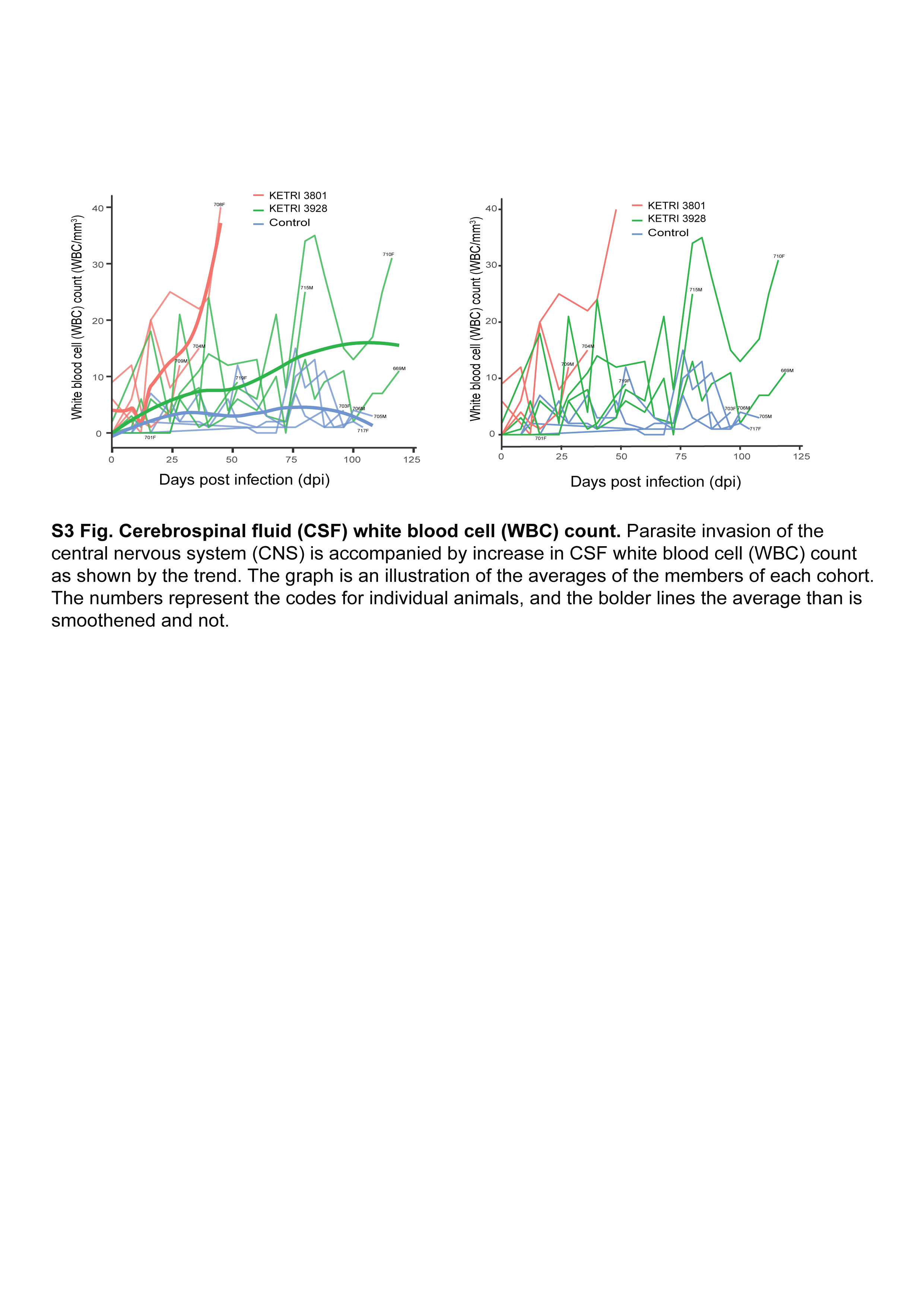

Supplement: Supplementary Text 1 — Ethical approval for animal use referenced C/TR/4/490/1. [file DataSheet1.zip › Figure S3.TIF]
